# Filth Diseases and Their Prevention

**Published:** 1876-07

**Authors:** 


### Books Reviewed.

*Filth Diseases and their Prevention.* By John Simon, M. D., F. R. C. S. Reprinted under the direction of the State Board of Health of Massachusetts. Boston: James Campbell, 1876.

This little work appeared first as the preface to a volume of reports made by Government Inspectors upon several epidemics which had occurred in various parts of England. It is so admirably suited to general perusal that the Massachusetts State Board of Health, through a committee, present it to the American public and urge its careful perusal. On page six the estimate is made that of the half million deaths which are yearly registered in England, fully one hundred and twenty-five thousand could be prevented if the existing knowledge of the chief causes of disease as affecting masses of the population were more reasonably well applied. The essay shows that local, and in most instances, preventable causes, have much to do with the death rate of various portions of England. For instance, in certain districts the death-rate of infants in the first year of life ranges from eight to twelve per cent. while in a still larger proportion of districts it ranges from nineteen to thirty per cent. Taking the death-rate of all ages it is found to be in England twenty-two and one-half per thousand per annum, which includes local rates ranging on the one hand from thirteen to seventeen, and on the other far above thirty per thousand.

In a large proportion of these enormous variations in the death-rate, filth in one form or another is directly chargeable with the production of disease, and it is with the view of enlightening the public upon the subject that this admirable essay has been issued by the State Board of Health of Massachusetts. Its careful study is properly urged upon all, that they may form a more intelligent estimate of the value of hyg ene as a conservator of public health. In this connection we make the following very pertinent extracts from the *Medical Record* on the "Hygiene of Ruralizing:"

"At this time of the year, when half the population of our cities are ruralizing, a great many questions concerning the strictly sanitary advantages of the practice present themselves for consideration. The physician, when he advises his patients to go to the country, does not as often as he might appreciate the responsibility which he assumes. There are so many qualifying conditions to be taken into account in the choice of a locality; that it is not surprising that many mistakes are made; that patients, instead of improving, get worse; indeed, that the very change which was thought to be beneficial had a direct influence in hastening their dissolution. Some of the disastrous effects of this advice cannot be very well avoided in the absence of exact knowledge concerning the effects of climate on disease; but there are other drawbacks which are preventable and remediable, and to which, as medical men, we should give our attention.

"We are of the opinion that there is not enough care taken to guard against the causes of disease which are now so generally recognized as associated with the unsanitary rural retreats. The time has gone by when the mountain side, the river shore, or the ocean beach is a synonym of health, and that a hap-hazard guess at the salubrity of the localities is always attended with good results. It has become the habit, however, with some medical men to consult the conveniencies of their patients as regards localities, rather than rely upon a positive knowledge of the claims of the said locality as a health resort.

"We have had within the past few years numerous examples of sickness in some of our most fashionable summer hotels, due to the neglect of the most ordinary sanitary regulations. When we consider this possibility in connection with large and expensive hotels, supplied 'with all the modern conveniences,' how much more will such a possibility of danger be exaggerated in the numerous farm-houses tinkered into the appearance of comfort and health, and overcrowded in the hottest time of the year by temporary boarders. The practitioners in rural districts have readily offered their testimony against these abominations, or so-called country boarding-houses; and numerous cases of preventable diseases occurring in them have come to our notice, due not only to overcrowding, but to culpable neglect of drainage, foul drinking-water, and other disease-engendering influences. So common is this condition of things, that it is becoming the exception rather than the rule to find these farm-houses even decently supplied with sanitary conveniences. When we add to this the risk of contamination of drinking-water, the frequent possibility of which has of late been so painfully impressed upon us, we become aware of the fact that a country retreat, far from being a health resort, may be the positive source of diseases which do not exist in the cities.

"We have repeatedly alluded to the dangerous condition of some of the religious camping-grounds, so largely occupied during the warmer months. Although much has been done to prevent disease, much more is required before we can be assured that the longer these grounds are occupied the more dangerously saturated will the soil become. It was only a short time ago that provisions were made to guard against the sewage saturation of one of the largest of these camping-grounds. Until then, drinking-water was obtained by the sinking of pipes in a sandy beach, while holes in the sand were the only receptables of the excrement. It is impossible to estimate how many cases of disease and death have been prevented by these means, the timely adoption of which seems almost providential. But many of these pilgrim pic-nic grounds are not in such a desirable condition, and the breaking out of camp fever, diarrhœal and other diseases, seems to be the merest question of time. The same remark applies with equal force and in a proportionate degree to many of the farm-houses, whose drains are badly cared for, and whose crowded condition intensify the risks of speedy soil contamination.

